# Blood–brain barrier vulnerability and microcirculatory dysfunction as predictors of hemorrhagic transformation after endovascular treatment in acute ischemic stroke

**DOI:** 10.3389/fnagi.2026.1783952

**Published:** 2026-04-20

**Authors:** Yuchen Wang, Qiujin Du, Wenjie Zhu, Zhang Yang, Yuanyuan Zhang

**Affiliations:** 1 Department of Neurology, Affiliated Hospital of Guizhou Medical University, Guiyang, Guizhou, China; 2 Department of Neurology, Affiliated Hospital of Nantong University, Nantong, Jiangsu, China

**Keywords:** acute ischemic stroke, blood-brain barrier vulnerability, endovascular treatment, hemorrhagic transformation, microcirculatory dysfunction, neuroinflammation

## Abstract

**Background:**

Hemorrhagic transformation (HT) after endovascular treatment (EVT) for acute ischemic stroke (AIS) remains a major clinical challenge, particularly in populations vulnerable to vascular and endothelial injury. Beyond conventional clinical predictors, microcirculatory dysfunction and blood–brain barrier (BBB) vulnerability may critically influence the risk of hemorrhagic complications following reperfusion. However, existing prediction models often rely on isolated imaging markers or basic clinical variables, failing to capture the integrated pathophysiological processes linking collateral circulation, microvascular perfusion, and BBB integrity.

**Methods:**

This retrospective study analyzed 202 consecutive AIS patients undergoing EVT (2021–2024). Collateral circulation was assessed via ASITN/SIR scale; CT perfusion (CTP)-derived parameters (rCBF, rCBV, rMTT, rTTP, rPS) quantified microcirculation and BBB integrity. Clinical/laboratory variables (including serum globulin) were collected. Multivariate logistic regression identified HT/parenchymal hematoma (PH) predictors; a nomogram was validated via ROC, calibration, bootstrap, DCA, and CIC. HT subtypes were correlated with functional outcomes.

**Results:**

HT occurred in 82 patients (40.6%, 37 PH cases). Higher ASITN/SIR grade (OR = 0.616), rCBF (OR = 0.053), and rCBV (OR = 0.204) were protective; rPS (OR = 2.624) and serum globulin (OR = 1.138) were risk factors for HT. The multimodal model (integrating three mechanistic pathways) showed excellent discrimination (AUC = 0.867) and superior net clinical benefit. For PH, ASITN/SIR was protective (AUC = 0.745). HT patients had poorer outcomes (*p* < 0.001), with PH worse than HI (*p* = 0.046).

**Conclusion:**

This study demonstrates that BBB vulnerability, microcirculatory dysfunction, and collateral status are key determinants of hemorrhagic transformation after endovascular treatment in acute ischemic stroke. By integrating these interrelated vascular and endothelial mechanisms, the proposed model provides insight into susceptibility to reperfusion-related hemorrhagic injury and offers a practical framework for individualized risk stratification in the peri-procedural setting.

## 1 Introduction

According to the Global Burden of Disease Study 2023, stroke remains the second leading cause of death worldwide (GBD 2023 Causes of Death Collaborators, 2025). As a major chronic non-communicable disease, stroke continues to be the primary cause of disability and mortality in China, with its annual incidence steadily increasing, posing a significant threat to public health (Tu et al., 2023). Acute ischemic stroke (AIS) occurs due to focal interruption of cerebral blood flow resulting from various etiologies, leading to neuronal injury through the ischemic cascade and subsequent local neurological deficits (Wang P. et al., 2024). In recent years, reperfusion therapy has become the cornerstone of AIS management, including intravenous thrombolysis and endovascular treatment (EVT) (Ho and Powers, 2025). EVT has emerged as the standard of care for large-vessel occlusion (LVO) stroke, as it rapidly restores blood flow to ischemic brain tissue, significantly improving recanalization rates and functional outcomes (Sheth, 2023). However, hemorrhagic transformation (HT) remains one of the most challenging complications following EVT (Sharma and Smith, 2022). The occurrence of HT not only increases mortality but also delays the initiation of antithrombotic and rehabilitation therapies. Therefore, the accurate identification of high-risk patients for HT prior to or during EVT remains a critical issue in acute stroke management. Although several studies have explored the predictive value of collateral circulation and CT perfusion (CTP) for HT, there is still a lack of integrative models combining these imaging factors with clinical indicators to enable clinically applicable predictions. This study, therefore, aims to develop a comprehensive predictive model that incorporates collateral circulation, CTP, and clinical factors.

The mechanisms underlying HT are multifactorial, involving ischemia–hypoxia, blood–brain barrier (BBB) disruption (Arba et al., 2021), inflammatory responses (Diestro et al., 2022), ischemia–reperfusion injury (IRI) (Binder et al., 2024; Pensato et al., 2025), coagulation dysfunction (Ye et al., 2020), and mechanical vascular injury during EVT (Teng et al., 2015). From a pathophysiological perspective, assessing cerebral microcirculation perfusion and BBB status, in conjunction with laboratory indices such as coagulation and inflammation, is of particular importance. Previous studies have primarily focused on clinical and laboratory predictors of HT, such as the HAT score, which evaluates post-thrombolytic bleeding risk based on NIHSS, blood glucose, and early CT changes (Lou et al., 2008). However, these parameters provide limited insights into cerebral microcirculation and BBB integrity. Collateral circulation acts as a compensatory blood supply to the ischemic penumbra during vessel occlusion, playing a crucial role in determining tissue viability and susceptibility to reperfusion injury (Deng et al., 2023). Currently, digital subtraction angiography (DSA) is the most widely used imaging modality for evaluating collateral circulation by dynamically visualizing vascular filling. It is considered the gold standard for grading secondary and tertiary collateral circulation (Fukuda and Liebeskind, 2023). Collateral status is commonly assessed using the American Society of Interventional and Therapeutic Neuroradiology/Society of Interventional Radiology (ASITN/SIR) scale (Hassen et al., 2019). In contrast, CTP provides both quantitative and visual assessments of cerebral perfusion and metabolic function (Kargiotis et al., 2023). CTP-derived parameters—such as cerebral blood flow (CBF), cerebral blood volume (CBV), mean transit time (MTT), and time to peak (TTP)—serve as important hemodynamic markers to delineate the ischemic penumbra and infarct core, and to define the therapeutic window (Kim et al., 2022; Sarraj et al., 2024). Additionally, parameters such as the volume transfer constant (K*^trans^*) and permeability–surface area product (PS) reflect BBB permeability (Arba et al., 2020; Xu et al., 2023), providing objective evidence for predicting reperfusion-related HT after AIS.

Multiple interrelated pathophysiological mechanisms likely contribute to HT after EVT in AIS, particularly impaired collateral circulation, microcirculatory dysfunction, and BBB vulnerability. However, these domains are often assessed separately in existing studies. Therefore, the present study aimed to integrate imaging and laboratory markers reflecting these complementary pathways into a unified predictive framework for individualized risk stratification after EVT.

## 2 Materials and methods

### 2.1 Patient enrollment

**FIGURE 1:**
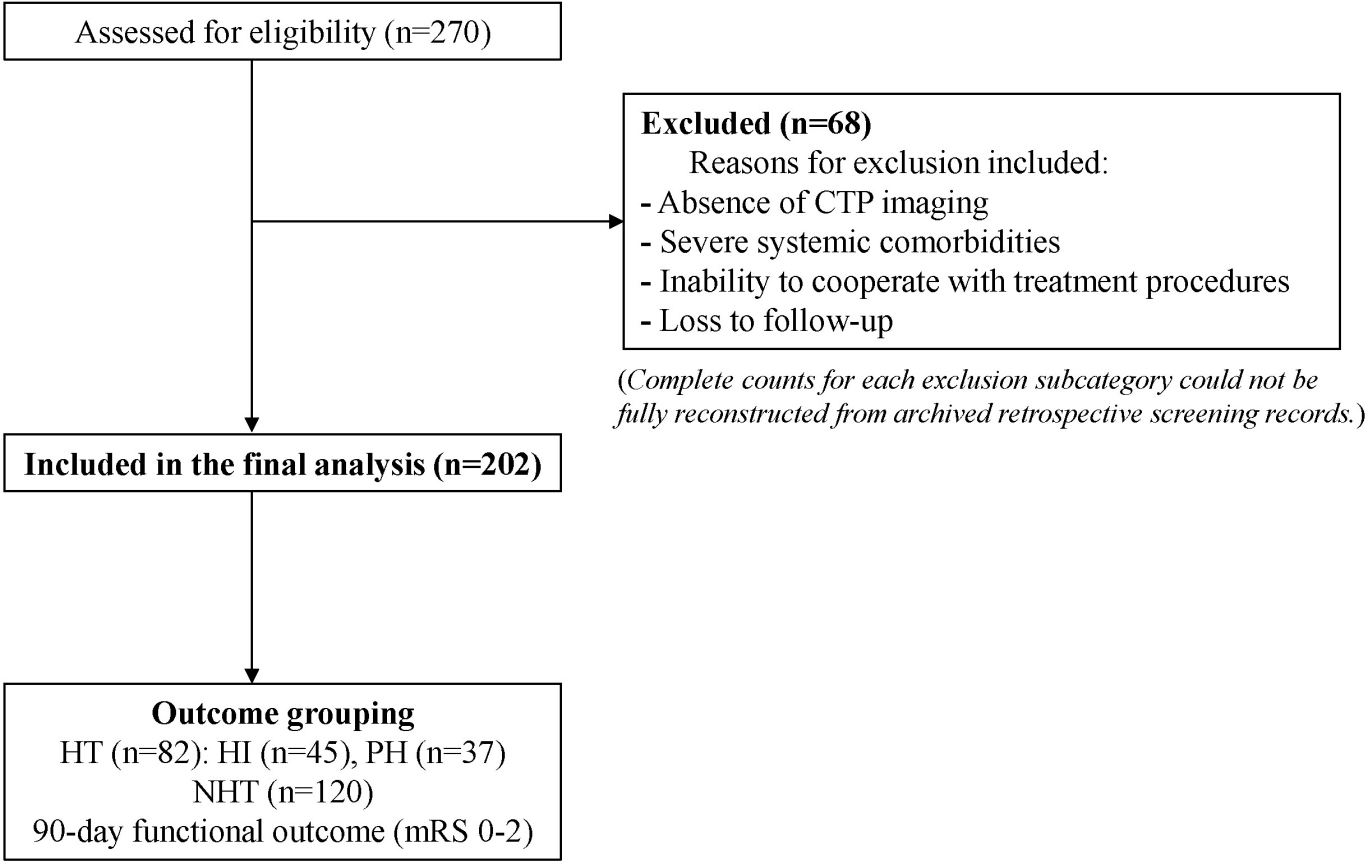
Flow diagram of patient screening, inclusion, and outcome grouping.

This study was a single-center retrospective cohort study that included consecutive AIS patients who underwent EVT at the Affiliated Hospital of Nantong University between September 2021 and September 2024. The sample size was calculated with α = 0.05 and β = 0.2, aiming for an expected area under the receiver operating characteristic (ROC) curve (AUC) ≥ 0.80. The minimum required sample size was 180, and a total of 202 patients were ultimately enrolled to ensure statistical validity. All study procedures adhered to the principles of medical ethics and the Declaration of Helsinki, and the study protocol was approved by the Ethics Committee of the Affiliated Hospital of Nantong University. During the study period, 68 patients were excluded because of absence of CTP imaging, severe systemic comorbidities, inability to cooperate with treatment procedures, or loss to follow-up. Owing to the retrospective nature of case screening, complete counts for every exclusion subcategory could not be fully reconstructed from the archived screening records. To avoid introducing potentially inaccurate retrospective estimates, only the overall exclusion count and verifiable screening information were reported. The detailed patient selection process is illustrated in Figure 1.

### 2.2 Inclusion and exclusion criteria

Inclusion criteria were as follows: (1) Age ≥ 18 years; (2) Diagnosis consistent with AIS criteria (Liu et al., 2020); (3) Underwent non-contrast CT (NCCT), CT angiography (CTA), and CTP within 24 h of admission; (4) Received EVT followed by postoperative CT or magnetic resonance imaging (MRI) reexamination; (5) Availability of complete clinical and follow-up data.

Exclusion criteria included: (1) Hemorrhagic stroke or stroke with a confirmed hemorrhagic etiology; (2) Imaging artifacts that interfered with analysis; (3) Severe systemic diseases affecting long-term prognosis, including but not limited to advanced hepatic or renal failure, terminal malignancy (life expectancy < 6 months), severe diabetic complications (e.g., ketoacidosis, diabetic foot), and advanced cardiovascular diseases (e.g., end-stage heart failure, severe coronary artery disease); (4) Inability to cooperate with treatment procedures due to severe cognitive impairment, agitation, or unstable clinical condition; (5) Incomplete clinical data or loss to follow-up. Patients classified as “loss to follow-up” were those without available follow-up imaging or clinical outcome assessment during hospitalization.

### 2.3 Clinical and laboratory data collection

Demographic and admission data were collected, including age, sex, medical and family history, medications, blood pressure, Trial of Org 10172 in Acute Stroke Treatment (TOAST) classification (Adams et al., 1993), and National Institutes of Health Stroke Scale (NIHSS) score (Brott et al., 1989) at admission (Supplementary Table 1). Laboratory indices included routine blood tests, C-reactive protein, hepatic and renal function tests, glucose metabolism and electrolytes, troponin I, and coagulation parameters. EVT-related data included bridging therapy, general anesthesia, puncture-to-recanalization time, thrombectomy techniques and balloon angioplasty.

### 2.4 Imaging acquisition protocol and parameters

CT perfusion (CTP) imaging was performed with a temporal sampling interval of 1 second per frame on a Siemens SOMATOM Force dual-source CT scanner. This high temporal resolution conforms to recommended standards for reliable deconvolution and accurate estimation of perfusion and permeability parameters, including CBF, CBV, MTT, TTP, and PS. CTP datasets were post-processed using Philips IntelliSpace Portal to generate quantitative perfusion maps, and relative parameters (rCBF, rCBV, rMTT, rTTP, and rPS) were calculated as the ratio between the affected and contralateral hemispheres. Early ischemic changes were assessed using the Alberta Stroke Program Early CT Score (ASPECTS) (Barber et al., 2000; Puetz et al., 2008), with scoring details for anterior and posterior circulation provided in Supplementary Tables 2, 3. Collateral circulation was evaluated using the American Society of Interventional and Therapeutic Neuroradiology/Society of Interventional Radiology (ASITN/SIR) grading system (Higashida et al., 2003), with details in Supplementary Table 4. Two experienced neuroradiologists independently evaluated all images while blinded to clinical data. Any discrepancies were resolved by consensus with a senior radiologist.

To ensure the reliability of subjective imaging assessments, two experienced neuroradiologists (with 5 and 7 years of specialized neuroimaging diagnostic experience, respectively) independently evaluated the ASITN/SIR grade and ASPECTS score in a blinded manner to all clinical and outcome data. Intra-observer agreement was assessed by having each radiologist re-evaluate 30 randomly selected cases 4 weeks later, without access to their initial assessments. Weighted kappa (κ) statistics were used to quantify agreement (accounting for the ordinal nature of the scoring systems), and the results showed excellent inter-observer agreement: ASITN/SIR grade [κ = 0.83, 95% confidence interval (CI): 0.75–0.91] and ASPECTS score (κ = 0.81, 95% CI: 0.72–0.90). Intra-observer agreement was also excellent: ASITN/SIR grade (κ = 0.87, 95% CI: 0.80–0.94) and ASPECTS score (κ = 0.85, 95% CI: 0.77–0.93), consistent with the Landis-Koch classification (κ > 0.80 indicates almost perfect agreement) (Landis and Koch, 1977). Any initial discrepancies between the two radiologists were resolved by consensus with a senior neuroradiologist (10 years of neuroimaging experience).

### 2.5 Outcome measures

The primary outcome was the occurrence of HT during hospitalization, while the secondary outcome was parenchymal hematoma (PH) and 90-days functional outcomes. HT was determined by follow-up cranial CT or MRI during hospitalization after EVT, and patients were divided into the HT and non-HT (NHT) groups. Based on the European Cooperative Acute Stroke Study (ECASS) classification (Hacke et al., 1995), HT was further categorized into hemorrhagic infarction (HI) and PH subtypes. Functional outcomes were evaluated using the modified Rankin Scale (mRS) at 90 days (Swieten et al., 1988). A good outcome is defined as 90-day mRS score 0–2 points. Detailed classification criteria are provided in Supplementary Tables 5, 6.

### 2.6 Statistical analysis

All statistical analyses were performed using SPSS version 27.0 and R version 4.2. Continuous variables were tested for normality using the Kolmogorov–Smirnov test. Normally distributed data were presented as mean ± standard deviation (x̄ ± s) and compared using the independent-samples *t*-test; non-normally distributed data were expressed as median (interquartile range) and compared using the Mann–Whitney U test. Categorical variables were expressed as number (percentage) and compared using the χ^2^-test.

Candidate predictors for multivariable logistic regression were first identified from variables associated with hemorrhagic transformation in univariate analyses. To avoid a purely data-driven approach, the final model was determined with additional consideration of clinical relevance, pathophysiological plausibility, and model parsimony. A combined predictive model and nomogram were constructed based on significant variables, and internal validation was performed using the bootstrap method. The model’s discrimination was assessed via the ROC curve and AUC; calibration was evaluated by the Hosmer–Lemeshow goodness-of-fit test and calibration curve. Decision curve analysis (DCA) and clinical impact curve (CIC) were further used to evaluate the clinical utility of the model. Functional outcomes were compared using the χ^2^ test. In the present cohort, 82 HT events occurred. With 5 predictors in the final model, the events-per-variable (EPV) ratio was approximately 16, exceeding the recommended minimum of 10 for stable logistic regression.

## 3 Results

### 3.1 Baseline data analysis

A total of 202 patients with AIS who underwent EVT were included; 137 were men (67.8%) and 65 were women (32.2%), and the mean age was 68.23 ± 9.80 years. Of these, 82 patients (40.6%) experienced HT: 45 had HI and 37 had PH. Based on clinical characteristics, significant differences were observed between the HT and NHT groups in TOAST classification, NIHSS score at admission, monocyte count, globulin, D-dimer, prothrombin time (PT), international normalized ratio (INR), thrombin time (TT), fibrin degradation products (FDP), troponin I, and balloon angioplasty (*p <* 0.05). The proportion of patients undergoing balloon angioplasty was significantly lower in the HT group compared to the NHT group (*p <* 0.05). Additionally, analysis of TOAST subtypes revealed that cardioembolic stroke was more common in the HT group than in the NHT group.

**FIGURE 2:**
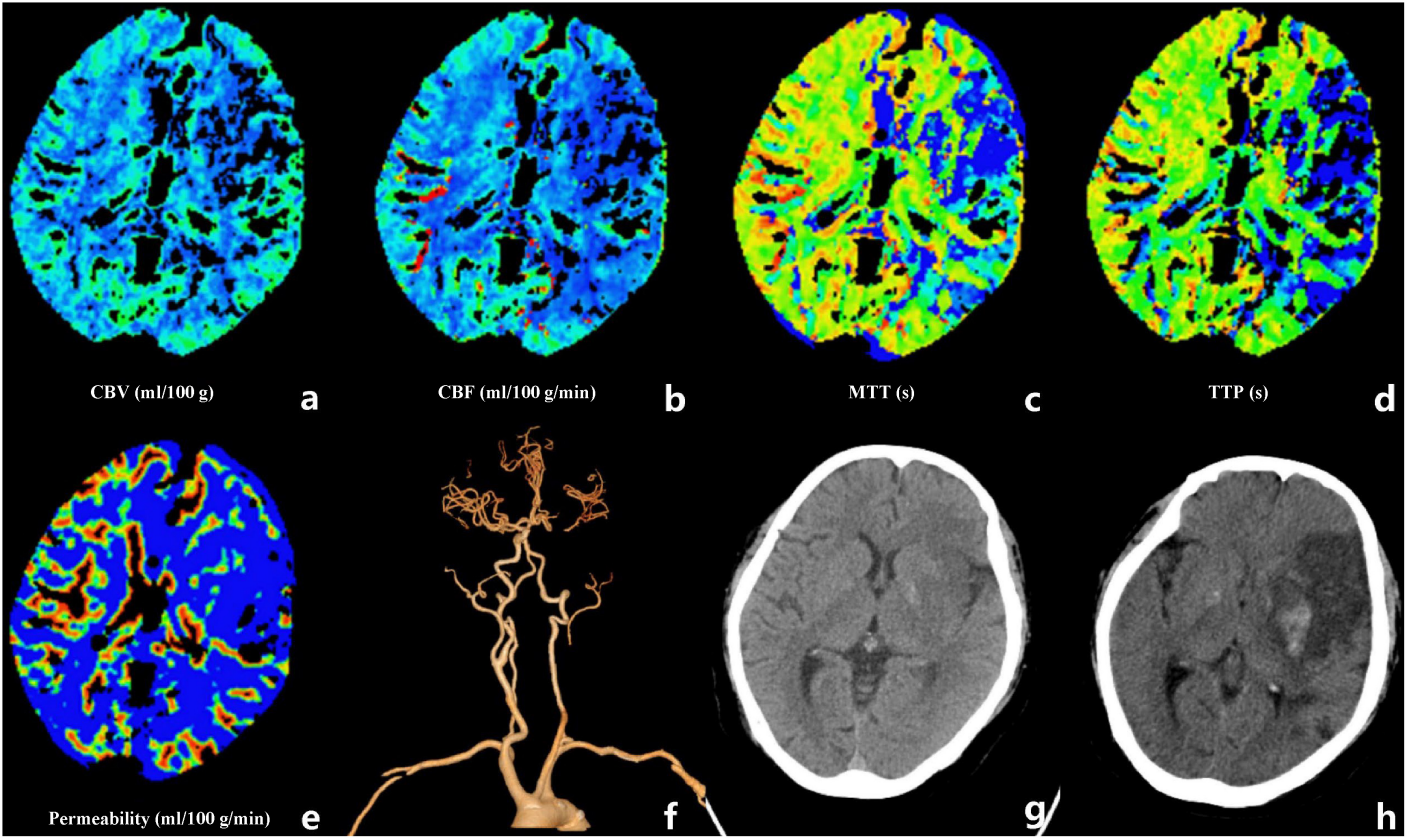
A representative case illustrating BBB vulnerability and microcirculatory dysfunction preceding HT after EVT in AIS. Baseline CTP maps demonstrate reduced CBF and CBV, with prolonged MTT and TTP in the affected hemisphere **(a–e)**. Notably, PS is markedly increased, indicating BBB vulnerability prior to reperfusion. Reconstructed CTA images confirm occlusion of the left internal carotid artery and middle cerebral artery **(f)**. Pre-treatment NCCT shows no obvious parenchymal hypodensity **(g)**, whereas follow-up NCCT obtained 24 h after EVT demonstrates hemorrhagic transformation within the infarcted region **(h)**. CTP, CT perfusion; CBF, cerebral blood flow; CBV, cerebral blood volume; MTT, mean transit time; TTP, time to peak; PS, permeability–surface area product; CTA, CT angiography; EVT, endovascular treatment; NCCT, non-contrast CT.

Imaging analysis showed that ASPECTS was significantly lower in the HT group (*p <* 0.001), consistent with more extensive parenchymal injury. Furthermore, rCBV and rCBF were significantly lower in the HT group (*p <* 0.001), whereas the rPS was significantly higher (*p <* 0.001). A representative HT case is shown in Figure 2, and a representative NHT case is shown in Supplementary Figure 1. The ASITN/SIR grade was also significantly associated with HT. Lower grades, indicating poorer collateralization, were strongly associated with higher HT incidence. Detailed relevant baseline characteristics are summarized in Table 1. More detailed baseline information can be found in Supplementary Table 7.

**TABLE 1:** Baseline characteristics of 202 patients with AIS undergoing EVT.

Variables	HT (*n* = 82)	NHT (*n* = 120)	t/χ ^2^/z	*p*
Age (year)	68.51 ± 8.69	68.04 ± 10.53	0.334	0.739
Male, n(%)	54 (65.9)	83 (69.2)	0.245	0.621
TOAST, n(%)			8.257	0.016
LAA	50(61.0)	92(76.7)		
CE	31 (37.8)	24 (20.0)		
SOE	1 (1.2)	4 (3.3)		
NIHSS	14 (10,20)	11 (6,17)	–3.203	0.001
Monocytes ( × 10^9^/L)	0.35 (0.27,0.54)	0.42 (0.32,0.66)	–2.788	0.005
Globulin (g/L)	30.87 ± 4.00	29.27 ± 4.39	2.641	0.009
D-dimer (mg/L)	0.91 (0.48,2.56)	0.61 (0.30,1.33)	–2.785	0.005
PT(s)	11.9 (11.3,12.5)	12.4 (11.8,13.0)	–3.391	<0.001
INR	1.06 (1.01,1.11)	1.02 (0.97,1.08)	–3.226	0.001
TT(s)	16.7 (15.7,17.4)	17.1 (16.3,17.9)	–2.138	0.032
FDP (μg/L)	3.13 (2.50,6.70)	2.50 (2.50,4.15)	–2.450	0.014
Troponin I (ng/mL)	0.012 (0.012,0.018)	0.012 (0.012,0.012)	–2.894	0.004
Balloon dilatation, n(%)	12 (14.6)	39 (32.5)	8.238	0.004
ASPECTS	6 (4,8)	8 (6,9)	–4.302	<0.001
ASITN/SIR	2 (1,3)	3 (2,3)	–4.925	<0.001
rCBV	0.38 (0.16,0.71)	0.71 (0.40,0.85)	–4.877	<0.001
rCBF	0.32 (0.18,0.49)	0.64 (0.39,0.87)	–5.699	<0.001
rMTT	2.56 (1.90,3.13)	2.27 (1.40,2.84)	–2.256	0.024
rTTP	1.61 (1.24,2.61)	1.45 (1.20,1.98)	–0.696	0.486
rPS	2.16 (1.47,3.48)	1.45 (1.20,1.98)	–5.521	<0.001

LAA, Large-artery atherosclerosis; CE, Cardioembolism; SOE, Other determined etiology; NIHSS, National Institutes of Health Stroke Scale; PT, prothrombin time; INR, international normalized ratio; TT, thrombin time; FDP, Fibrin Degradation Products; ASPECTS, Alberta Stroke Program Early CT Score; ASITN/SIR, American Society of Interventional and Therapeutic Neuroradiology/ Society of Interventional Radiology.

During the study period, 270 patients with AIS undergoing EVT were screened. A total of 68 patients were excluded. Because screening was retrospective, complete counts for each exclusion subcategory could not be fully reconstructed from the archived screening records. The final analysis included 202 patients, of whom 82 developed HT (45 HI and 37 PH) and 120 did not develop HT. CTP, computed tomography perfusion; HT, hemorrhagic transformation; HI, hemorrhagic infarction; PH, parenchymal hematoma; mRS, modified Rankin Scale.

### 3.2 Multivariate logistic regression analysis for HT

Multivariate logistic regression analysis identified globulin (OR = 1.138, 95% CI: 1.027–1.260, *p* = 0.013) and rPS (OR = 2.719, 95% CI: 1.689–4.378, *p* < 0.001) as independent risk factors for HT. Conversely, ASITN/SIR grade (OR = 0.641, 95% CI: 0.414–0.993, *p* = 0.046), rCBV (OR = 0.204, 95% CI: 0.051–0.816, *p* = 0.003), and rCBF (OR = 0.053, 95% CI: 0.011–0.268, *p* < 0.001) were identified as independent protective factors. The results are detailed in Table 2.

**TABLE 2:** Univariate and multivariate logistic regression analysis of predictors for HT post-EVT.

Variables	OR	95% CI	*p*
TOAST	0.637	0.268–1.514	0.307
Admission NIHSS	1.037	0.983–1.093	0.180
Neutrophils	0.839	0.582–1.208	0.344
Globulin (g/L)	1.138	1.027–1.260	0.013
D-dimer (mg/L)	1.012	0.659–1.554	0.957
PT	1.450	0.215–9.779	0.703
INR	0.124	0.0–1179904.1	0.843
TT	0.840	0.624–1.130	0.249
FDP	0.976	0.800–1.191	0.813
Troponin I	1.255	0.619–2.544	0.529
Balloon dilatation	0.573	0.207–1.584	0.283
ASPECTS	0.904	0.734–1.114	0.345
ASITN/SIR grade	0.641	0.414–0.993	0.046
rCBV	0.204	0.051–0.816	0.003
rCBF	0.053	0.011–0.268	<0.001
rMTT	0.229	0.049–1.079	0.062
rPS	2.719	1.689–4.378	<0.001

### 3.3 Construction of the nomogram prediction model

**FIGURE 3:**
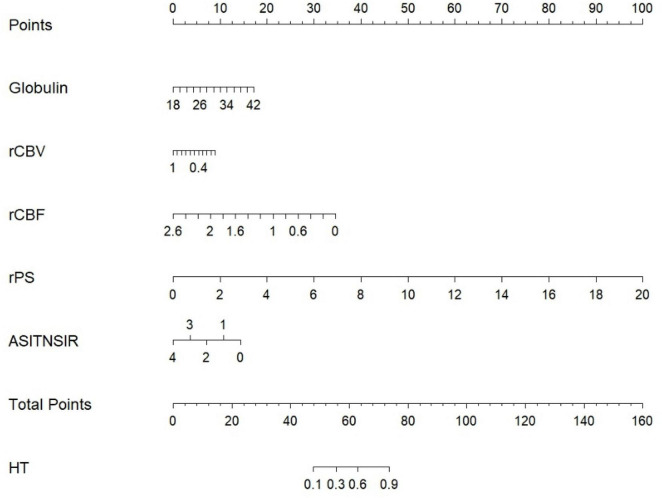
Nomogram integrating collateral circulation (ASITN/SIR Grade), CTP (rCBV, rCBF, rPS), and globulin for predicting post-EVT HT Risk.

Five independent predictors—globulin, ASITN/SIR grade, rCBV, rCBF, and rPS—were incorporated into a multivariate logistic regression model. The β coefficients used for model construction are shown in Supplementary Table 8. The final predictive equation was formulated as: logit (P) = –2.655 + 0.122 × (Globulin) -1.522 × (rCBV) - 2.256 × (rCBF) + 0.849 × (rPS) - 0.609 × (ASITN/SIR). The probability of HT after EVT was calculated as: P = e^[logit(P)]/(1 + e^[logit(P)]). A corresponding nomogram was constructed using R software to visualize the risk contribution of each variable (Figure 3). The total point range was 0–160, where: 0–17 points corresponded to globulin levels between 18 and 42 g/L; 0–100 points corresponded to rPS values between 0 and 20; 0–8 points corresponded to rCBV values between 1 and 0.4; 0–35 points corresponded to rCBF values between 2.6 and 0; 0–15 points corresponded to ASITN/SIR grades between 4 and 0. When the total score ranged between 48 and 73, the predicted probability of HT after EVT ranged from 10 to 90%.

### 3.4 Model validation and evaluation

**FIGURE 4:**
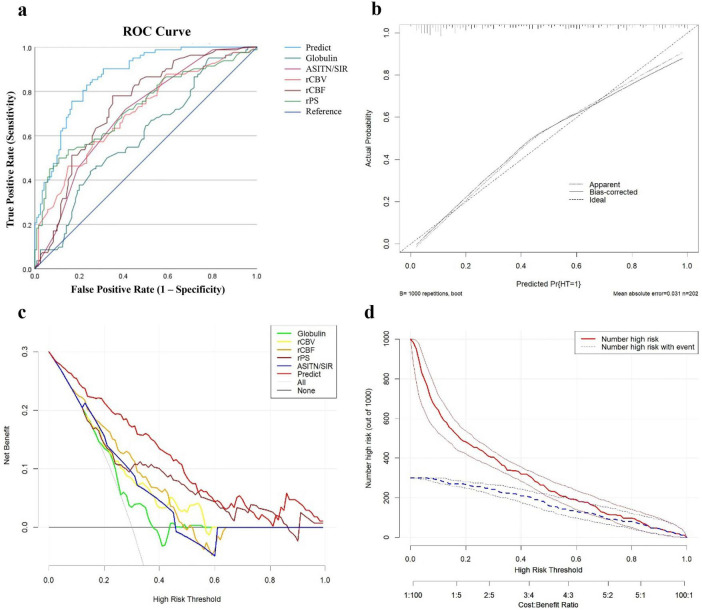
Performance evaluation of the integrated model for HT risk: **(a)** receiver operating characteristic (ROC) curves; **(b)** calibration curve; **(c)** decision curve analysis (DCA); and **(d)** clinical impact curve (CIC).

The predictive performance of each predictor (globulin, rCBV, rCBF, rPS, and ASITN/SIR grade) and the integrated predictive model was compared using ROC curve analysis (Figure 4a). The combined model exhibited the highest predictive efficacy, with AUC = 0.867 (*p <* 0.001), sensitivity (Se) = 0.829, and specificity (Sp) = 0.767. At an rCBF threshold of 0.495, AUC was 0.736 (*p <* 0.001), with Se = 0.650 and Sp = 0.780. The discriminative ability of individual parameters in descending order was: rPS, rCBV, ASITN/SIR, and globulin (Supplementary Table 9). Internal validation via bootstrap resampling yielded a C-statistic and calibration AUC of 0.8675, confirming excellent discriminatory ability (∼86.75%) between HT and NHT patients. The Hosmer–Lemeshow goodness-of-fit test (χ^2^ = 8.8943, df = 8, *p* = 0.3513) and calibration curve (Figure 4b) demonstrated good model calibration. Furthermore, decision curve analysis (DCA) and clinical impact curve (CIC) (Figures 4c,d) confirmed that the integrated model achieved higher net clinical benefit than “treat-all” or “treat-none” strategies when the risk threshold exceeded 1%. Within a threshold probability range of 1–70%, the model maintained stable high net benefit. When the predicted probability exceeded 65%, the model’s identification of high-risk HT patients closely matched the actual incidence, indicating strong clinical effectiveness.

### 3.5 Imaging analysis of HT subtypes

Among the 82 patients with HT after EVT, 45 cases were classified as HI and 37 as PH according to the ECASS criteria. Comparative analysis revealed that ASPECTS score, ASITN/SIR grade, and rPS significantly differed between the HI and PH groups (*p <* 0.05; Table 3). Multivariate logistic regression analysis including these three variables showed that ASITN/SIR grade (OR = 0.521, 95% CI: 0.280–0.968, *p* = 0.039) was an independent protective factor against PH, whereas ASPECTS and rPS were not independently associated with PH. The discriminative ability of ASITN/SIR grading for predicting PH was further evaluated using ROC curve analysis. The results showed that the AUC of ASITN/SIR grading was 0.745 (*p* < 0.001, Se 0.622, Sp 0.711).

**TABLE 3:** Imaging features of different HT subtypes.

Variables	HI (*n* = 45)	PH (*n* = 37)	z	*p*
ASPECTS	7 (5, 8)	4 (3, 5)	–4.229	<0.001
ASITN/SIR grade	2 (1, 3)	1 (0, 2)	–3.941	<0.001
rCBV	0.42 (0.15, 0.76)	0.32 (0.20, 0.65)	–0.210	0.834
rCBF	0.37 (0.20, 0.49)	0.30 (0.12, 0.48)	–1.165	0.244
rMTT	2.46 (1.68, 1.99)	2.62 (1.99, 3.12)	–0.704	0.482
rTTP	1.55 (1.25, 2.88)	1.64 (1.19, 2.53)	–0.368	0.713
rPS	1.67 (1.35, 2.76)	2.68 (2.03, 3.61)	–2.661	0.008

#### 3.6 Functional outcome analysis of HT and PH subtypes

**FIGURE 5:**
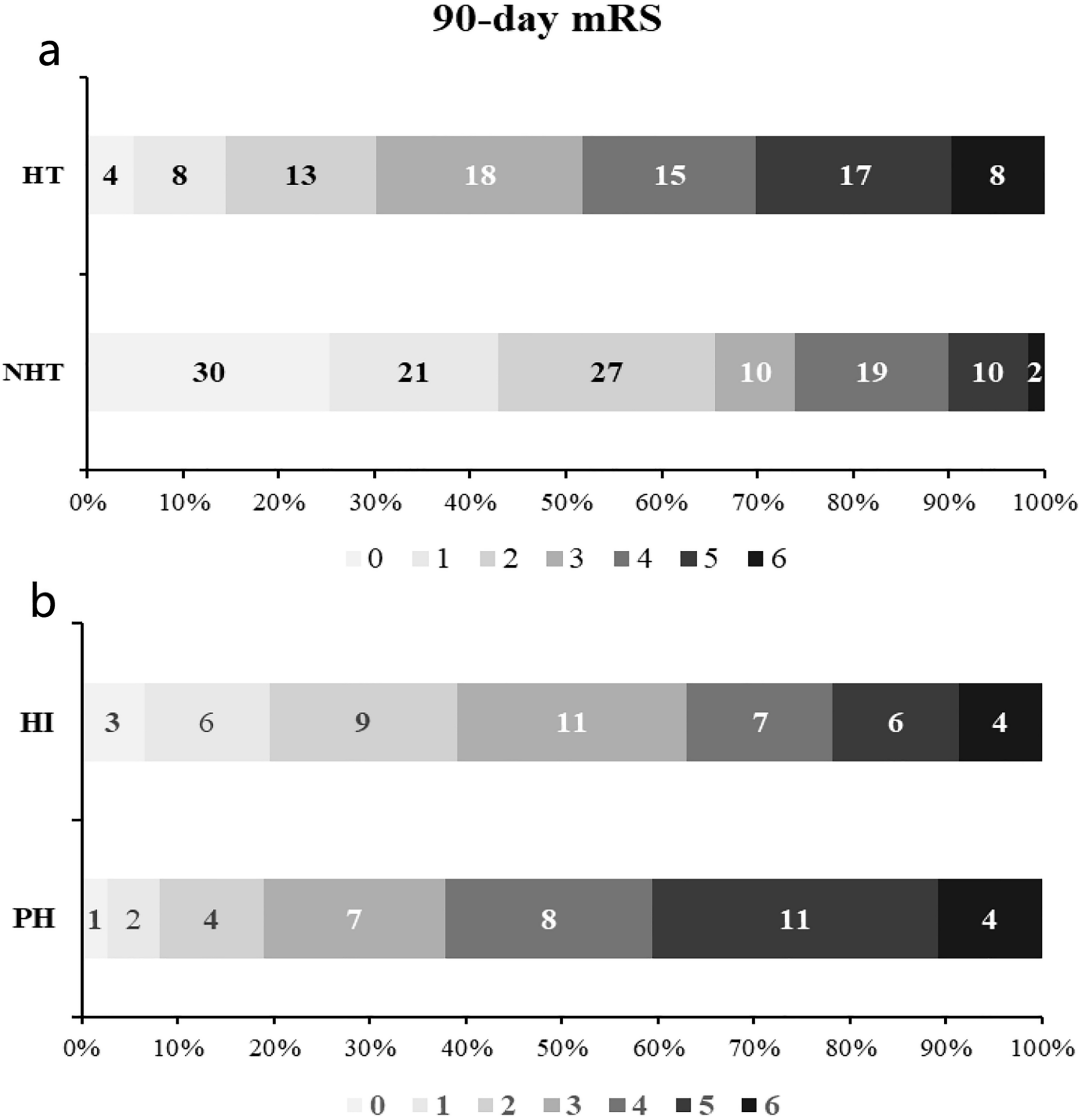
Distribution of 90-day modified Rankin Scale (mRS) scores. **(a)** HT status (HT vs. NHT); **(b)** hemorrhagic subtypes (HI vs. PH).

We summarized the composition ratio of the 90 day mRS scores of 202 patients (Figure 5). The proportion of patients achieving a good outcome (90-day mRS 0–2) differed significantly between the HT and NHT groups. Patients with HT had a markedly lower rate of good functional outcome compared with those without HT (χ^2^ = 24.555, *p* < 0.001), indicating a clear association between HT and poor long-term prognosis. Among patients with HT, functional outcomes also differed between subtypes. The proportion of good outcome was significantly lower in the PH group than in the HI group (χ^2^ = 3.980, *p* = 0.046). This finding suggests that PH, representing the more severe form of HT, is associated with a higher likelihood of unfavorable functional outcome at 90 days compared with HI.

## 4 Discussion

HT following EVT represents a complex interplay between reperfusion dynamics and intrinsic vascular vulnerability of the ischemic brain. In this study, we demonstrate that BBB vulnerability, microcirculatory dysfunction, and collateral status jointly determine the risk of HT after EVT in AIS. Rather than acting as isolated predictors, these factors reflect interconnected pathophysiological processes governing endothelial integrity, capillary perfusion, and tissue resilience during reperfusion.

Importantly, BBB permeability, quantified by rPS, emerged as an independent risk factor for HT, highlighting the central role of endothelial dysfunction in mediating reperfusion-related injury. Experimental and clinical evidence suggests that endothelial dysfunction, impaired autoregulation, and heightened inflammatory responses may collectively exacerbate BBB vulnerability under ischemia-reperfusion stress (Alaqel et al., 2025). Concurrently, impaired microcirculatory perfusion, reflected by reduced rCBF and rCBV, may amplify local hemodynamic instability and promote capillary rupture in susceptible tissue (Wang P. et al., 2024). Collateral circulation further modulates this vulnerability by sustaining perfusion and mitigating abrupt reperfusion pressure. Together, these findings support a mechanistic framework in which HT arises from the convergence of vascular and endothelial susceptibility during reperfusion, providing insight into individualized risk stratification beyond conventional clinical predictors.

ASITN/SIR grading, as a key indicator of collateral circulation, reflects the brain’s compensatory capacity during arterial occlusion. Robust collaterals sustain perfusion in the ischemic penumbra, mitigating reperfusion pressure peaks, capillary rupture, and subsequent HT risk. Consistent with prior evidence, patients with good collaterals exhibit lower HT incidence and mortality post-EVT (Liu et al., 2025); a systematic meta-analysis of 18 clinical studies further corroborates this (Sinha et al., 2023). Additionally, reductions in rCBF and rCBV indicate microcirculatory impairment and tissue viability decline, correlating with elevated HT risk. Our results align with Lakhani et al. (2025), who reported rCBV < 42% as an independent predictor of HT. Although thresholds differ slightly from prior studies (Lakhani et al., 2025; McDonough et al., 2024), the association of reduced rCBV and rCBF with HT remains consistently reported across multiple investigations. Similarly, a systematic review and meta-analysis of 12 clinical studies showed that early venous filling (EVF) during EVT signals cerebral hyperperfusion and vascular injury, increasing HT risk (Lima et al., 2025). Elevated rPS reflects increased BBB permeability and endothelial dysfunction, indicating enhanced hemorrhagic propensity (Liu et al., 2018). Beyond PS, other imaging markers for BBB permeability include K*^trans^*, extraction fraction, and hypoperfusion intensity ratio (Abualnadi et al., 2025; Bivard et al., 2020). PS’s advantage lies in its direct derivation from standard post-processing software, without extra computations, enhancing clinical feasibility. Future studies should compare these parameters’ relative performance, which is one of our planned directions. Elevated serum globulin emerged as an independent predictor of HT in our analysis. Total serum globulin constitutes a heterogeneous mixture of plasma proteins, including α−, β−, and γ−globulins (Hoo et al., 2021). Although γ−globulins (immunoglobulins) have been linked to inflammatory activation and BBB disruption (Carmona-Mora et al., 2023), and some globulin fractions may influence coagulation and fibrinolysis (Nezu et al., 2013), total globulin itself does not specifically reflect inflammatory activity. Therefore, the observed association should not be interpreted as evidence of a direct causal mechanism, but rather as a surrogate of broader systemic inflammatory, prothrombotic, or physiological disturbances. Accordingly, this finding should be interpreted with caution, and further studies incorporating more specific inflammatory markers or globulin subfractions are warranted to elucidate the underlying biological pathways.

Collectively, these independent predictors converge on three interrelated physiological pathways: (1) collateral compensation; (2) microcirculation perfusion; (3) BBB permeability. The integrated comprehensive prediction model demonstrated high discriminative performance (AUC = 0.867; Se = 0.829; Sp = 0.767), surpassing single variables (AUC = 0.601–0.736). Hosmer-Lemeshow tests and calibration curves confirmed good model calibration, while DCA and CIC indicated significant net clinical benefit. Prior models often relied on single imaging markers (Busto et al., 2025; Chen et al., 2019; Mokin et al., 2015), with limited predictive accuracy. Although recent CTP-based nomograms predict post-thrombolysis HT (Zheng et al., 2025), similar models for EVT-related HT remain absent. To address this gap, we provide a logistic regression–based nomogram that enables quantitative estimation of hemorrhagic risk while preserving mechanistic interpretability across collateral, microcirculatory, and BBB-related pathways. The model yields positive net benefit at low threshold probabilities ( ≥ 1%), underscoring its potential for early perioperative screening. Its conceptual coherence—integrating macro-vascular supply, micro-vascular perfusion, and BBB integrity—not only enhances physiological interpretability but also amplifies clinical decision value, offering a quantitative integrated assessment of HT risk.

Notably, 90-day mRS outcome analysis showed HT was significantly associated with reduced functional independence (χ^2^ = 24.555, *p* < 0.001), with PH exerting far greater impact than HI (χ^2^ = 3.980, *p* = 0.046). These results are similar to those of a multicenter analysis that compared to HI, PH carries higher mortality and worse clinical outcomes, especially PH2 (Honig et al., 2022). It also highlights the profound clinical implications of HT subtypes. We found that lower ASITN/SIR grading and higher rPS strongly correlated with PH occurrence, with ASITN/SIR grading as an independent protective factor for PH. This aligns with prior research, where good collaterals sustain continuous perfusion in the ischemic region, reducing severe bleeding risks like PH2 (Lan et al., 2025). Our multimodal prognostic model demonstrated favorable discrimination for predicting HT incidence and may assist in subtype-oriented risk assessment, particularly through the prognostic value of ASITN/SIR grading for PH. By quantifying interactions between collateral deficits and perfusion injury, the model supports targeted interventions, such as personalized antithrombotic regimens and subtype-guided rehabilitation, ultimately alleviating long-term disability and optimizing post-EVT recovery trajectories. Clinically, high-risk phenotypes (collateral-poor, lowest rCBF/rCBV, highest rPS) warrant vigilance for hemodynamics, cautious antithrombotics, and continuous neuroimaging monitoring—principles validated by DCA/CIC prediction-observation concordance. Importantly, the variables incorporated in the model are available at clinically relevant time points in centers where CTP is included in the routine EVT workflow, without requiring substantial additional procedures. Importantly, the model is not intended to replace established EVT eligibility criteria or to delay reperfusion therapy in otherwise eligible patients. Rather, it may serve as a peri-procedural risk stratification tool to identify patients at increased risk of HT after reperfusion. In clinical practice, such patients may benefit from closer hemodynamic monitoring, more individualized blood pressure control, cautious initiation of antithrombotic therapy, and intensified early neuroimaging follow-up. At the current stage, however, the nomogram should be regarded as a supportive assessment tool rather than a fixed-threshold decision model, and clinically actionable cutoff values will require further external validation.

Several limitations should be acknowledged. First, the single-center retrospective design may introduce selection bias and residual confounding; notably, mandatory CTP imaging for study inclusion further contributes to selection bias given that CTP is not uniformly performed in all acute ischemic stroke patients, and exclusion of patients with severe systemic comorbidities may additionally limit the generalizability of our findings to routine clinical populations. In addition, because patient screening was performed retrospectively, complete counts for every exclusion subcategory could not be fully reconstructed from the archived screening records, which may have limited the granularity of screening transparency. Although the sample size was adequate for internal validation, it limits generalizability to broader populations. Second, CTP parameters may vary across scanners and post-processing software. Although using relative values mitigates systematic bias, multicenter prospective studies with standardized acquisition protocols are needed to establish universally applicable thresholds. Moreover, even with a 1-s temporal sampling interval consistent with current recommendations, differences in vendor-specific post-processing algorithms may still lead to inter-platform variability in quantitative perfusion parameters. Third, imaging assessments were confined primarily to baseline and early follow-up time points, lacking longitudinal dynamic monitoring. Finally, concurrent collection of BBB permeability or inflammatory biomarkers was absent, limiting imaging-molecular data integration and mechanistic inference. Future research should adopt prospective multicenter designs, standardize imaging acquisition and post-processing protocols to ensure cross-platform consistency, and incorporate longitudinal imaging with complementary molecular biomarkers. Although standardized Picture Archiving and Communication Systems (PACS)-based imaging infrastructure is available in many tertiary stroke centers, external validation across diverse populations remains essential before broader clinical generalization.

## 5 Conclusion

This retrospective study identified ASITN/SIR grade, rCBF, and rCBV as independent protective factors against HT after EVT for acute ischemic stroke, whereas elevated rPS emerged as an independent risk factor. In addition, collateral status was independently protective against PH. Building on these findings, we developed a multidimensional integrative model centered on collateral compensation, microcirculatory perfusion, and BBB vulnerability. By unifying heterogeneous imaging and clinical indicators within a coherent pathophysiological framework, the model provides quantitative estimates of hemorrhagic risk and demonstrates superior discriminative performance compared with individual predictors, including for clinically important subtypes such as HT and PH. Importantly, this integrative approach offers insight into vascular and endothelial susceptibility to reperfusion-related injury and supports individualized risk stratification across the peri-procedural course of EVT. These findings underscore the potential value of mechanism-informed imaging markers for guiding personalized management strategies and informing future translational research on reperfusion-related vascular injury.

## Data Availability

The original contributions presented in this study are included in the article/Supplementary material, further inquiries can be directed to the corresponding authors.

## References

[B1] Abualnadi Y. D. Miller S. Khalil Z. M. Sadiq K. O. S. Tekle W. G. Hassan A. E. (2025). On old dogs and new tricks: CT perfusion predicts hemorrhagic transformation after thrombectomy. *Interv. Neuroradiol.* 10.1177/15910199251386104 [Epub ahead of print]. 41066500 PMC12510989

[B2] Adams H. P. Bendixen B. H. Kappelle L. J. Biller J. Love B. B. Gordon D. L. et al. (1993). Classification of subtype of acute ischemic stroke. Definitions for use in a multicenter clinical trial. TOAST. Trial of org 10172 in acute stroke treatment. *Stroke* 24 35–41. 10.1161/01.str.24.1.35 7678184

[B3] Alaqel S. I. Imran M. Khan A. Nayeem N. (2025). Aging, vascular dysfunction, and the blood-brain barrier: Unveiling the pathophysiology of stroke in older adults. *Biogerontology* 26:67. 10.1007/s10522-025-10209-y 40044939

[B4] Arba F. Piccardi B. Palumbo V. Biagini S. Galmozzi F. Iovene V. et al. (2021). Blood-brain barrier leakage and hemorrhagic transformation: The Reperfusion Injury in Ischemic StroKe (RISK) study. *Eur. J. Neurol.* 28 3147–3154. 10.1111/ene.14985 34143500

[B5] Arba F. Rinaldi C. Caimano D. Vit F. Busto G. Fainardi E. (2020). Blood-brain barrier disruption and hemorrhagic transformation in acute ischemic stroke: Systematic review and meta-analysis. *Front. Neurol.* 11:594613. 10.3389/fneur.2020.594613 33551955 PMC7859439

[B6] Barber P. A. Demchuk A. M. Zhang J. Buchan A. M. (2000). Validity and reliability of a quantitative computed tomography score in predicting outcome of hyperacute stroke before thrombolytic therapy. ASPECTS Study Group. Alberta Stroke Programme Early CT Score. *Lancet* 355 1670–1674. 10.1016/s0140-6736(00)02237-6 10905241

[B7] Binder N. F. Amki M. E. Glück C. Middleham W. Reuss A. M. Bertolo A. et al. (2024). Leptomeningeal collaterals regulate reperfusion in ischemic stroke and rescue the brain from futile recanalization. *Neuron* 112 1456–1472.e6. 10.1016/j.neuron.2024.01.031 38412858

[B8] Bivard A. Kleinig T. Churilov L. Levi C. Lin L. Cheng X. et al. (2020). Permeability measures predict hemorrhagic transformation after ischemic stroke. *Ann. Neurol.* 88 466–476. 10.1002/ana.25785 32418242 PMC7496077

[B9] Brott T. Adams H. P. Olinger C. P. Marler J. R. Barsan W. G. Biller J. et al. (1989). Measurements of acute cerebral infarction: A clinical examination scale. *Stroke* 20 864–870. 10.1161/01.str.20.7.864 2749846

[B10] Busto G. Morotti A. Casetta I. Danesi F. Loverre F. Casseri T. et al. (2025). Refining the Tmax malignant profile in large ischemic core patients receiving endovascular treatment. *Eur. J. Radiol.* 189:112187. 10.1016/j.ejrad.2025.112187 40408913

[B11] Carmona-Mora P. Knepp B. Jickling G. C. Zhan X. Hakoupian M. Hull H. et al. (2023). Monocyte, neutrophil, and whole blood transcriptome dynamics following ischemic stroke. *BMC Med.* 21:65. 10.1186/s12916-023-02766-1 36803375 PMC9942321

[B12] Chen W. Song X. Tian D. Sun H. Zhang L. Hui X. et al. (2019). Clinical efficacy of collateral circulation in the evaluation of endovascular treatment for acute internal carotid artery occlusion. *Heliyon* 5:e01476. 10.1016/j.heliyon.2019.e01476 31008404 PMC6458472

[B13] Deng G. Chu Y.-H. Xiao J. Shang K. Zhou L.-Q. Qin C. et al. (2023). Risk factors, pathophysiologic mechanisms, and potential treatment strategies of futile recanalization after endovascular therapy in acute ischemic stroke. *Aging Dis.* 14 2096–2112. 10.14336/ad.2023.0321-1 37199580 PMC10676786

[B14] Diestro J. D. B. Parra-Farinas C. Balas M. Zador Z. Almusalam N. Dmytriw A. A. et al. (2022). Inflammatory biomarkers and intracranial hemorrhage after endovascular thrombectomy. *Can. J. Neurol. Sci.* 49 644–650. 10.1017/cjn.2021.197 34548113

[B15] Fukuda K. A. Liebeskind D. S. (2023). Evaluation of collateral circulation in patients with acute ischemic stroke. *Radiol. Clin. North Am.* 61 435–443. 10.1016/j.rcl.2023.01.002 36931760

[B16] GBD 2023 Causes of Death Collaborators (2025). Global burden of 292 causes of death in 204 countries and territories and 660 subnational locations, 1990-2023: A systematic analysis for the Global Burden of Disease Study 2023. *Lancet* 406 1811–1872. 10.1016/s0140-6736(25)01917-8 41092928 PMC12535838

[B17] Hacke W. Kaste M. Fieschi C. Toni D. Lesaffre E. Kummer R. V. et al. (1995). Intravenous thrombolysis with recombinant tissue plasminogen activator for acute hemispheric stroke. The European Cooperative Acute Stroke Study (ECASS). *JAMA* 274 1017–1025. 10.1001/jama.1995.03530130023023 7563451

[B18] Hassen W. B. Malley C. Boulouis G. Clarençon F. Bartolini B. Bourcier R. et al. (2019). Inter- and intraobserver reliability for angiographic leptomeningeal collateral flow assessment by the American Society of Interventional and Therapeutic Neuroradiology/Society of Interventional Radiology (ASITN/SIR) scale. *J. Neurointerv. Surg.* 11 338–341. 10.1136/neurintsurg-2018-014185 30131382

[B19] Higashida R. T. Furlan A. J. Roberts H. Tomsick T. Connors B. Barr J. et al. (2003). Trial design and reporting standards for intra-arterial cerebral thrombolysis for acute ischemic stroke. *Stroke* 34 e109–e137. 10.1161/01.Str.0000082721.62796.09 12869717

[B20] Ho J. P. Powers W. J. (2025). Contemporary management of acute ischemic stroke. *Annu. Rev. Med.* 76 417–429. 10.1146/annurev-med-050823-094312 39496213

[B21] Honig A. Molad J. Horev A. Simaan N. Sacagiu T. Figolio A. et al. (2022). Predictors and prognostic implications of hemorrhagic transformation following cerebral endovascular thrombectomy in acute ischemic stroke: A multicenter analysis. *Cardiovasc. Intervent. Radiol.* 45 826–833. 10.1007/s00270-022-03115-0 35296934

[B22] Hoo T. Lim E. M. John M. D’Orsogna L. McLean-Tooke A. (2021). Calculated globulin as a screening tool for hypogammaglobulinaemia or paraproteins in hospitalized patients. *Ann. Clin. Biochem.* 58 236–243. 10.1177/0004563221989737 33430600

[B23] Kargiotis O. Psychogios K. Safouris A. Andrikopoulou A. Eleftheriou A. Spiliopoulos S. et al. (2023). Computed tomography perfusion imaging in acute ischemic stroke: Accurate interpretation matters. *Stroke* 54 e104–e108. 10.1161/strokeaha.122.041117 36756889

[B24] Kim B. K. Kim B. You S.-H. (2022). Clinical relevance of computed tomography perfusion-estimated infarct volume in acute ischemic stroke patients within the 6-h therapeutic time window. *Cerebrovasc. Dis.* 51 438–446. 10.1159/000519901 35066495

[B25] Lakhani D. A. Balar A. B. Ali S. Khan M. Salim H. A. Koneru M. et al. (2025). The relative cerebral blood volume (rCBV)<42% is independently associated with hemorrhagic transformation in anterior circulation large vessel occlusion. *Interv. Neuroradiol.* 10.1177/15910199241308322 [Epub ahead of print]. 39763336 PMC11705296

[B26] Lan Z. Zheng J. Zhang X. Zhang J. Chen Z. Chen Y. et al. (2025). Enhancing prediction of parenchymal hemorrhage type 2 after endovascular treatment in acute ischemic stroke using dual-phase CTA. *Eur. J. Radiol.* 186:112027. 10.1016/j.ejrad.2025.112027 40043546

[B27] Landis J. R. Koch G. G. (1977). The measurement of observer agreement for categorical data. *Biometrics* 33 159–174. 10.2307/2529310 843571

[B28] Lima N. L. Mezzari M. H. D. S. Júnior L. C. I. C. Grüdtner D. O. M. Maggi B. G. Azevedo D. K. D. et al. (2025). Early venous filling following reperfusion therapy in acute ischemic stroke: A systematic review and meta-analysis. *Neuroradiology* 10.1007/s00234-025-03722-x [Epub ahead of print]. 40748454

[B29] Liu C. Yan S. Zhang R. Chen Z. Shi F. Zhou Y. et al. (2018). Increased blood-brain barrier permeability in contralateral hemisphere predicts worse outcome in acute ischemic stroke after reperfusion therapy. *J. Neurointerv. Surg.* 10 937–941. 10.1136/neurintsurg-2017-013663 29352054

[B30] Liu G. Zhu H. Li L. Yang J. Shi X. Yang S. et al. (2025). Effectiveness of collateral status on clinical outcomes in patients with established large infarct: A prospective cohort study. *Int. J. Surg.* 111 9342–9352. 10.1097/js9.0000000000003162 40844268 PMC12695354

[B31] Liu L. Chen W. Zhou H. Duan W. Li S. Huo X. et al. (2020). Chinese Stroke Association guidelines for clinical management of cerebrovascular disorders: Executive summary and 2019 update of clinical management of ischaemic cerebrovascular diseases. *Stroke Vasc. Neurol.* 5 159–176. 10.1136/svn-2020-000378 32561535 PMC7337371

[B32] Lou M. Safdar A. Mehdiratta M. Kumar S. Schlaug G. Caplan L. et al. (2008). The HAT Score: A simple grading scale for predicting hemorrhage after thrombolysis. *Neurology* 71 1417–1423. 10.1212/01.wnl.0000330297.58334.dd 18955684 PMC2676961

[B33] McDonough R. V. Rex N. B. Ospel J. M. Kashani N. Rinkel L. A. Sehgal A. et al. (2024). Association between CT perfusion parameters and hemorrhagic transformation after endovascular treatment in acute ischemic stroke: Results from the ESCAPE-NA1 trial. *Am. J. Neuroradiol.* 45 887–892. 10.3174/ajnr.A8227 38697793 PMC11286015

[B34] Mokin M. Morr S. Fanous A. A. Shallwani H. Natarajan S. K. Levy E. I. et al. (2015). Correlation between cerebral blood volume values and outcomes in endovascular therapy for acute ischemic stroke. *J. Neurointerv. Surg.* 7 705–708. 10.1136/neurintsurg-2014-011279 25147229

[B35] Nezu T. Hosomi N. Aoki S. Deguchi K. Masugata H. Ichihara N. et al. (2013). Alpha2-macroglobulin as a promising biomarker for cerebral small vessel disease in acute ischemic stroke patients. *J. Neurol.* 260 2642–2649. 10.1007/s00415-013-7040-x 23877435

[B36] Pensato U. Demchuk A. M. Menon B. K. Nguyen T. N. Broocks G. Campbell B. C. V. et al. (2025). Cerebral infarct growth: Pathophysiology, pragmatic assessment, and clinical implications. *Stroke* 56 219–229. 10.1161/strokeaha.124.049013 39545332

[B37] Puetz V. Sylaja P. N. Coutts S. B. Hill M. D. Dzialowski I. Mueller P. et al. (2008). Extent of hypoattenuation on CT angiography source images predicts functional outcome in patients with basilar artery occlusion. *Stroke* 39 2485–2490. 10.1161/strokeaha.107.511162 18617663

[B38] Sarraj A. Pujara D. K. Campbell B. C. (2024). Current state of evidence for neuroimaging paradigms in management of acute ischemic stroke. *Ann. Neurol.* 95 1017–1034. 10.1002/ana.26925 38606939

[B39] Sharma D. Smith M. (2022). The intensive care management of acute ischaemic stroke. *Curr. Opin. Crit. Care* 28 157–165. 10.1097/mcc.0000000000000912 35034076

[B40] Sheth S. A. (2023). Mechanical thrombectomy for acute ischemic stroke. *Continuum* 29 443–461. 10.1212/con.0000000000001243 37039404

[B41] Sinha A. Stanwell P. Killingsworth M. C. Bhaskar S. M. M. (2023). Prognostic accuracy and impact of cerebral collateral status on clinical and safety outcomes in acute ischemic stroke patients receiving reperfusion therapy: A systematic meta-analysis. *Acta Radiol.* 64 698–718. 10.1177/02841851221080517 35311387

[B42] Swieten J. C. V. Koudstaal P. J. Visser M. C. Schouten H. J. van Gijn J. (1988). Interobserver agreement for the assessment of handicap in stroke patients. *Stroke* 19 604–607. 10.1161/01.str.19.5.604 3363593

[B43] Teng D. Pannell J. S. Rennert R. C. Li J. Li Y.-S. Wong V. W. et al. (2015). Endothelial trauma from mechanical thrombectomy in acute stroke: In vitro live-cell platform with animal validation. *Stroke* 46 1099–1106. 10.1161/strokeaha.114.007494 25712942

[B44] Tu W.-J. Wang L.-D. Special Writing Group of China Stroke Surveillance Report (2023). China stroke surveillance report 2021. *Mil. Med. Res.* 10:33. 10.1186/s40779-023-00463-x 37468952 PMC10355019

[B45] Wang P. Konja D. Singh S. Zhang B. Wang Y. (2024). Endothelial senescence: From macro- to micro-vasculature and its implications on cardiovascular health. *Int. J. Mol. Sci.* 25:1978. 10.3390/ijms25041978 38396653 PMC10889199

[B46] Wang Y. Li M. Jiang Y. Ji Q. (2024). Comparative efficacy of neuroprotective agents for improving neurological function and prognosis in acute ischemic stroke: A network meta-analysis. *Front. Neurosci.* 18:1530987. 10.3389/fnins.2024.1530987 39834702 PMC11743486

[B47] Xu J. Dai F. Wang B. Wang Y. Li J. Pan L. et al. (2023). Predictive value of CT perfusion in hemorrhagic transformation after acute ischemic stroke: A systematic review and meta-analysis. *Brain Sci.* 13:156. 10.3390/brainsci13010156 36672136 PMC9856940

[B48] Ye C. Wang Y. Song Q. Liu J. Wei C. Liu M. (2020). Association between coagulation function and spontaneous hemorrhagic transformation in acute ischemic stroke. *Curr. Neurovasc. Res.* 17 344–353. 10.2174/1567202617666200514114258 32407276

[B49] Zheng Y. Jiang P. Xu X. Xue L. Chen J. Xue Y. (2025). A quantitative CT perfusion-derived online dynamic nomogram for predicting hemorrhagic transformation after intravenous thrombolysis in acute ischemic stroke. *Eur. J. Radiol. Open* 15:100685. 10.1016/j.ejro.2025.100685 41017931 PMC12465048

