# Supplementary material for: Blood–brain barrier vulnerability and microcirculatory dysfunction as predictors of hemorrhagic transformation after endovascular treatment in acute ischemic stroke
Source: Front Aging Neurosci. 2026 Apr 20;18:1783952. doi: 10.3389/fnagi.2026.1783952 (PMC13136286; doi:10.3389/fnagi.2026.1783952)
Supplement: Supplementary file 1 [file Data_Sheet_1.pdf]

Supplementary Table 1. National Institutes of Health Stroke Scale (NIHSS)

Category	Description	Score
1a. LOC: Alertness	0 = Alert, 1 = Not alert, 2 = Requires repeated stimulation, 3 = Coma	0–3
1b. LOC Questions	0 = Answers both correctly, 1 = One correct, 2 = None correct	0–2
1c. LOC Commands	0 = Obeys both, 1 = One correct, 2 = None correct	0–2
2. Best Gaze	0 = Normal, 1 = Partial palsy, 2 = Forced deviation	0–2
3. Visual Fields	0 = No loss, 1 = Partial hemianopia, 2 = Complete hemianopia, 3 = Bilateral blindness	0–3
4. Facial Palsy	0 = Normal, 1 = Minor paralysis, 2 = Partial paralysis, 3 = Complete paralysis	0–3
5. Motor Arm	0 = No drift, 1 = Drift, 2 = Effort against gravity, 3 = No effort, 4 = No movement	0–4 each side
6. Motor Leg	Same as Motor Arm criteria	0–4 each side
7. Limb Ataxia	0 = None, 1 = One limb, 2 = Two limbs	0–2
8. Sensory	0 = Normal, 1 = Mild loss, 2 = Severe loss	0–2
9. Best Language	0 = Normal, 1 = Mild aphasia, 2 = Severe aphasia, 3 = Mute/global aphasia	0–3
10. Dysarthria	0 = Normal, 1 = Mild, 2 = Severe	0–2
11. Extinction/Inattention	0 = Normal, 1 = Neglect in one modality, 2 = Severe neglect	0–2

Supplementary Table 2. Anterior Circulation ASPECTS Scoring

Scored region	Score
1. Caudate nucleus（C）	1
2. Lentiform nucleus（L）	1
3. Posterior limb of the internal capsule（IC）	1
4. Insular cortex（I）	1
5. Anterior MCA cortical region（M1）	1
6. MCA cortex lateral to the insula（M2）	1
7. Posterior MCA cortical region（M3）	1
8. MCA cortex superior to M1（M4）	1
9. MCA cortex superior to M2（M5）	1
10. MCA cortex superior to M5（M6）	1

*MCA: Middle Cerebral Artery.

Supplementary Table 3. Posterior Circulation ASPECTS Scoring

Scored region	Score
1. Any part of the pons	2
2. Any part of the midbrain	2
3. Left cerebellum	1
4. Right cerebellum	1
5. Left thalamus	1
6. Right thalamus	1
7. Left posterior cerebral artery territory	1
8. Right posterior cerebral artery territory	1

Supplementary Table 4. ASITN/SIR Collateral Circulation Grading

Grade	Description
0	No collaterals visible to the ischemic site
1	Slow collaterals to the periphery of the ischemic site with persistence of some of the defect
2	Rapid collaterals to the periphery of ischemic site with persistence of some of the defect and to only a portion of the ischemic territory
3	Collaterals with slow but complete angiographic blood flow of the ischemic bed by the late venous phase
4	Complete and rapid collateral blood flow to the vascular bed in the entire ischemic territory by retrograde perfusion

Supplementary Table 5. European Cooperative Acute Stroke Study (ECASS) Classification

Type	Description
HI1	Small isolated petechiae along the margins of the infarct
HI2	Confluent petechiae within the infarcted area
PH1	Blood clots in ≤30% of the infarct area with a slight space-occupying effect
PH2	Blood clots in >30% of the infarct area with a substantial space-occupying effect

* HI, Hemorrhagic Infarction; PH, Parenchymal Hemorrhage

Supplementary Table 6. Modified Rankin Scale (mRS) score

Grade	Description
0	No symptoms
1	No significant disability; able to carry out all usual activities despite symptoms
2	Slight disability; unable to carry out all previous activities but able to look after own affairs without assistance
3	Moderate disability; requiring some help, but able to walk without assistance
4	Moderately severe disability; unable to walk without assistance and unable to attend to own bodily needs without assistance
5	Severe disability; bedridden, incontinent, and requiring constant nursing care and attention
6	Death

Supplementary Table 7. Extended baseline characteristics of AIS patients undergoing EVT.

	HT (n=82)	NHT (n=120)	t/χ2/z	*p*
**Demographics**				
Male, n (%)	54(65.9)	83(69.2)	0.245	0.621
Age (years)	68.51±8.69	68.04±10.53	0.334	0.739
**Admission data**				
Hypertension, n (%)	46(56.1)	74(61.7)	0.627	0.429
Diabetes mellitus, n (%)	13(15.9)	31(25.8)	2.848	0.092
Hyperlipidemia, n (%)	4(4.9)	4(3.3)	0.306	0.580
Smoking history, n (%)	13(15.9)	29(24.2)	2.636	0.104
Admission SBP (mmHg)	149.43±25.56	143.18±20.42	1.924	0.056
Admission DBP (mmHg)	85.43±14.74	82.10±12.73	1.71	0.089
Culprit Vessel, n (%)			10.399	0.065
Internal carotid artery	31(37.8)	32(26.7)		
Middle cerebral artery	44(53.7)	58(48.3)		
Vertebral artery	1(1.2)	3(2.5)		
Basilar artery	1(1.2)	10(8.3)		
Posterior cerebral artery	5(6.1)	17(14.2)		
TOAST, n (%)			8.257	0.016*
LAA	50(61.0)	92(76.7)		
CE	31(37.8)	24(20.0)		
SOE	1(1.2)	4(3.3)		
Admission NIHSS	14(10,20)	11(6,17)	-3.203	0.001
**Laboratory Parameters**				
White blood cell ($\times10^9$/L)	7.40(5.89,9.53)	7.55(6.03,9.95)	-0.599	0.549
Neutrophils ($\times10^9$/L)	6.08(4.29,8.25)	6.28(4.17,9.41)	-0.984	0.325
Lymphocytes ($\times10^9$/L)	1.20(0.88,1.65)	1.39(0.90,2.39)	-1.923	0.054
Monocytes ($\times10^9$/L)	0.35(0.27,0.54)	0.42(0.32,0.66)	-2.788	0.005
Red blood cells ($\times10^{12}$/L)	4.47±0.21	5.16±1.04	-1.032	0.303
Hemoglobin (g/L)	140(126,150)	144.0(133,157)	-1.961	0.050
Platelets ($\times10^9$/L)	166(133,210)	182(143,219)	-1.735	0.083
C-reactive protein (mg/L)	4.04 (1.42,9.13)	2.51(1.04,6.71)	-1.963	0.050
ALT (U/L)	28(23,35)	26(22,33)	-1.210	0.226
AST (U/L)	22(18,28)	22(17,30)	-0.305	0.440
Total protein (g/L)	72.09±6.43	71.07±7.85	0.974	0.331
Albumin (g/L)	41.10±3.80	41.61±5.14	-0.815	0.416
Globulin (g/L)	30.87±4.00	29.27±4.39	2.641	0.009

	**HT (n=82)**	**NHT (n=120)**	**t/χ2/z**	***p***
Glucose (mmol/L)	7.3(6.2,8.2)	7.2(6.4,9.6)	-0.772	0.440
Creatinine (μmol/L)	63(52,75)	68(56,79)	-1.853	0.064
Uric acid (mmol/L)	340.12±105.72	351.88±103.94	-0.784	0.434
HbA1c (%)	5.8(5.4,6.8)	5.9(5.5,6.8)	-0.918	0.359
K^+ (mmol/L)	4.03±0.21	3.97±0.41	0.982	0.327
Na^+ (mmol/L)	139.87±3.56	139.86±3.41	-0.040	0.968
Cl^- (mmol/L)	116.60±11.02	113.90±3.89	1.043	0.300
Ca^{2+} (mmol/L)	2.26±0.15	2.27±0.12	-1.692	0.092
Phosphate (mmol/L)	1.15±0.20	1.15±0.24	-0.104	0.918
Mg^{2+} (mmol/L)	0.83±0.08	0.84±0.09	-1.159	0.248
D-dimer (mg/L)	0.91(0.48,2.56)	0.61(0.30,1.33)	-2.785	0.005
PT (s)	11.9(11.3,12.5)	12.4(11.8,13.0)	-3.391	＜0.001
APTT (s)	26.3(24.8,28.4)	27.0(25.0,28.8)	-0.718	0.473
Fibrinogen (g/L)	2.95(2.44,3.59)	2.88(2.40,3.47)	-0.456	0.648
INR	1.06(1.01,1.11)	1.02(0.97,1.08)	-3.226	0.001
TT (s)	16.7(15.7,17.4)	17.1(16.3,17.9)	-2.138	0.032
FDP (μg/L)	3.13(2.50,6.70)	2.50(2.50,4.15)	-2.450	0.014
Troponin I (ng/mL)	0.012(0.012,0.018)	0.012(0.012,0.012)	-2.894	0.004
**EVT-Related Data**				
Bridging therapy, n (%)	28(34.1)	50(41.7)	1.162	0.281
Puncture-to-recanalization time (min)	80.5(50,111)	90(55,112)	-0.050	0.960
Aspiration thrombectomy, n (%)	46(56.1)	54(45.0)	0.135	0.713
Stent retriever thrombectomy, n (%)	54(65.9)	76(63.3)	0.054	0.816
Balloon angioplasty, n (%)	12(14.6)	39(32.5)	8.238	0.004
**Imaging Parameters**				
ASPECTS	6(4,8)	8(6,9)	-4.302	＜0.001
ASITN/SIR grade	2(1,3)	3(2,3)	-4.925	＜0.001
rCBV	0.38(0.16,0.71)	0.71(0.40,0.85)	-4.877	＜0.001
rCBF	0.32(0.18,0.49)	0.64(0.39,0.87)	-5.699	＜0.001
rMTT	2.56(1.90,3.13)	2.27(1.40,2.84)	-2.256	0.024
rTTP	1.61(1.24,2.61)	1.45(1.20,1.98)	-0.696	0.486
rPS	2.16(1.47,3.48)	1.45(1.20,1.98)	-5.521	＜0.001

* SBP, systolic blood pressure; DBP, diastolic blood pressure; LAA, Large-artery atherosclerosis; CE, Cardioembolism; SOE, Other determined etiology; NIHSS, National Institutes of Health Stroke Scale; ALT, Alanine Aminotransferase; AST, Aspartate Aminotransferase; HbA1c,

Glycosylated Hemoglobin, Type A1C; K^+, potassium; Na^+, sodium; Cl^-, chlorine; Ca^{2+}, calcium; Mg^{2+}, magnesium; PT, prothrombin time; APTT, activated partial thromboplastin time; INR, international normalized ratio; TT, thrombin time; FDP, Fibrin Degradation Products; ASPECTS, Alberta Stroke Program Early CT Score; ASITN/SIR, American Society of Interventional and Therapeutic Neuroradiology/Society of Interventional Radiology.

Supplementary Table 8. Multivariate Binary Logistic Regression Analysis of Independent Predictors for HT After EVT in AIS

	β	Wald	*p*	OR	95% CI
Globulin	0.122	2.65	0.0081	1.924	1.186-3.121
rCBV	-1.522	-2.18	0.0290	0.428	0.200-0.917
rCBF	-2.256	-3.15	0.0016	0.292	0.136-0.629
rPS	0.849	4.15	＜0.0001	2.273	1.542-3.351
ASITN/SIR grade	-0.609	-3.72	0.0002	0.296	0.156-0.562
Constant	-2.655	-1.71	0.0867	1.550	

Supplementary Table 9. ROC analysis of Individual Predictors in Predicting HT Post-EVT

	AUC	*p*	Se	Sp	Yoden Index	Optimal threshold
ASITN/SIR grade	0.698	＜0.001	0.592	0.720	0.312	2.5
Globulin	0.601	0.015	0.439	0.75	0.189	31.85
rCBV	0.702	＜0.001	0.850	0.463	0.313	0.265
rCBF	0.736	＜0.001	0.650	0.780	0.430	0.495
rPS	0.729	＜0.001	0.500	0.892	0.392	2.170
Prediction model	0.867	＜0.001	0.829	0.767	0.596	0.381

* Se, sensitivity; Sp, specificity.

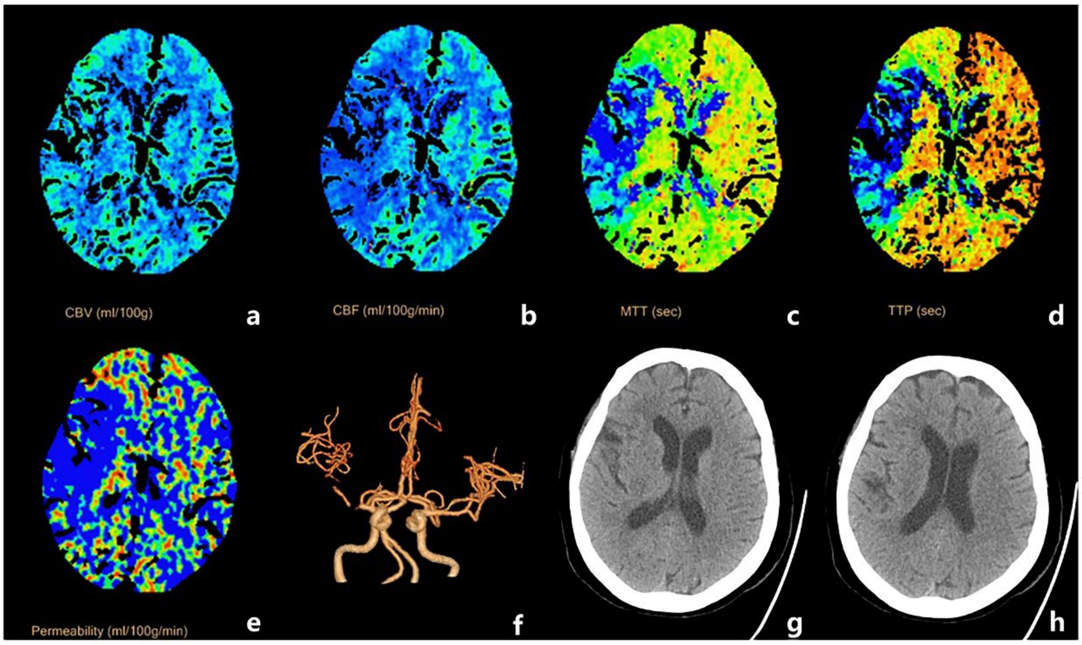

Supplementary Figure 1. A representative case without hemorrhagic transformation is shown for comparison, illustrating preserved blood–brain barrier integrity despite microcirculatory alterations on baseline CTP imaging. Baseline CTP demonstrates reduced CBF with relatively preserved CBV and no marked increase in PS (Supplementary Figures 1a–e). Reconstructed CTA images confirm occlusion of the right middle cerebral artery (Supplementary Figure 1f). Pre-treatment NCCT shows no obvious parenchymal hypodensity (Supplementary Figure 1g), follow-up NCCT obtained 24 hours after EVT shows no evidence of hemorrhagic transformation (Supplementary Figure 1h). CTP, CT perfusion; CBF, cerebral blood flow; CBV, cerebral blood volume; MTT, mean transit time; TTP, time to peak; PS, permeability–surface area product; CTA, CT angiography; EVT, endovascular treatment; NCCT, non-contrast CT.
